# Research and therapy with induced pluripotent stem cells (iPSCs): social, legal, and ethical considerations

**DOI:** 10.1186/s13287-019-1455-y

**Published:** 2019-11-21

**Authors:** Sharif Moradi, Hamid Mahdizadeh, Tomo Šarić, Johnny Kim, Javad Harati, Hosein Shahsavarani, Boris Greber, Joseph B. Moore

**Affiliations:** 1 Department of Stem Cells and Developmental Biology, Cell Science Research Center, Royan Institute for Stem Cell Biology and Technology, ACECR, Tehran, Iran; 2 Department of Cancer Medicine, Cell Science Research Center, Royan Institute for Stem Cell Biology and Technology, ACECR, Isar 11, 47138-18983, Babol, Iran; 3 Center for Physiology and Pathophysiology, Institute for NeurophysiologyMedical Faculty, University of Cologne, 50931 Cologne, Germany; 4 Department of Cardiac Development and Remodeling, Max-Planck-Institute for Heart and Lung Research, Bad Nauheim, Germany; 5 Laboratory of Regenerative Medicine and Biomedical Innovations, Pasteur Institute of Iran, Tehran, Iran; 6 Department of Cellular and Molecular Sciences, Faculty of Bioscience and Biotechnology, Shahid Beheshti University, Tehran, Iran; 7 RheinCell Therapeutics GmbH, 40764 Langenfeld, Germany; 8 Diabetes and Obesity Center, University of Louisville, Louisville, KY USA; 9 The Christina Lee Brown Envirome Institute, University of Louisville, Louisville, KY USA

**Keywords:** ELSI, Informed consent, SCNT, Cell manufacturing, GMP, ATMP, Clinical trial, Transgenic cells, Intellectual property, Tetraploid complementation

## Abstract

Induced pluripotent stem cells (iPSCs) can self-renew indefinitely in culture and differentiate into all specialized cell types including gametes. iPSCs do not exist naturally and are instead generated (“induced” or “reprogrammed”) in culture from somatic cells through ectopic co-expression of defined pluripotency factors. Since they can be generated from any healthy person or patient, iPSCs are considered as a valuable resource for regenerative medicine to replace diseased or damaged tissues. In addition, reprogramming technology has provided a powerful tool to study mechanisms of cell fate decisions and to model human diseases, thereby substantially potentiating the possibility to (i) discover new drugs in screening formats and (ii) treat life-threatening diseases through cell therapy-based strategies. However, various legal and ethical barriers arise when aiming to exploit the full potential of iPSCs to minimize abuse or unauthorized utilization. In this review, we discuss bioethical, legal, and societal concerns associated with research and therapy using iPSCs. Furthermore, we present key questions and suggestions for stem cell scientists, legal authorities, and social activists investigating and working in this field.

## Introduction

Induced pluripotent stem cells (iPSCs) are artificial stem cells produced from somatic cells through co-expression of defined pluripotency-associated factors [1, 2]. Like embryonic stem cells (ESCs), they can typically proliferate and self-renew indefinitely in vitro and differentiate into derivatives of all three primary germ layers (i.e., ectoderm, mesoderm, and endoderm) as well as germ cells that give rise to the gametes. However, according to the strictest definition, genuine or bona fide iPSCs could develop into an entire embryo in conjunction with extraembryonic membranes. Since the full pluripotency of iPSCs has been demonstrated by several studies through the most stringent test of pluripotency, i.e., tetraploid complementation (see Glossary), it is possible to derive truly pluripotent iPSCs from somatic cells [3–5]. Because of these features, iPSCs have numerous biomedical applications in basic research, drug screening, toxicological studies, disease modeling, and cell therapy [6]. Prior to 2007, PSCs from humans could only be derived from pre-implantation embryos such as morula- or blastocyst-stage embryos [7]. The resulting PSCs, known as ESCs, opened new avenues for research and perspectives for clinical practice. However, two important challenges have confined their broad application: [1] ethical limitations, since human embryos are destroyed to produce ESCs, and [2] immunological rejection of the cells differentiated from ESCs upon allogeneic cell transplantation [8]. Of note, the use of pre-implantation embryos for ESC derivation is not ethically challenging or legally banned in some countries including Canada, Sweden, Spain, France, Great Britain, Japan, Australia, Iran, and China, either because pre-implantation embryos are not considered to be “functional” human beings or their legislative bodies permit the creation or use of human embryos for research and therapeutic purposes [9]. In many other countries, however, the use of ESCs and/or ESC-derived cells is restricted or completely prohibited [10].

The basic paradigm in the use of PSCs for cell therapy purposes is that they are first differentiated into the desired cell types of interest, and the resulting specialized tissue-specific cells are then transplanted as cell suspensions or more complex tissue constructs into patients. The differentiation step is crucial because if proliferating, undifferentiated PSCs are directly injected, they would form tumors called teratomas due to their highly proliferative nature and broad differentiation potential [11–13]. When using ESCs as the source of differentiated cells, immunological issues remain a major challenge for cell therapy, in most parts because the donor cells most likely do not originate from the recipient patient [14]. For example, donor cells differentiated from ESCs express their own human leukocyte antigen (HLA) proteins which would be recognized as foreign by the recipient’s immune system if these cells are transplanted into a patient with a different HLA haplotype, leading to immune rejection of the cell graft. Simultaneous administration of immunosuppressive drugs can aid in overcoming these problems but can induce serious side effects. Therefore, two different approaches have been proposed to overcome the immunogenicity of ESC-derived grafts: establishment of ESC donor banks covering various HLAs from selected homozygous HLA-typed volunteers, or production of ESCs from patients themselves using somatic cell nuclear transfer (SCNT), which is also known as therapeutic cloning [15, 16]. The former strategy is labor-intensive, time-consuming, and expensive, because it would require HLA-typing of hundreds of thousands of individuals to establish a cell bank. Moreover, due to the inherent complexity of HLA biology and heterogeneity across individuals, it may not be possible to find HLA-matched ESCs for all subjects. However, only a relatively small number of samples would be required to match the large proportion of the population with a minimal requirement for immunosuppression. For example, calculations showed that only 150 selected homozygous HLA-typed cell lines could match 93% of the UK population [17] while 140 unique HLA homozygous donors would be needed to cover 90% of the Japanese population [18]. The latter approach harnessing SCNT enables to obtain blastocysts from any given patient and to produce the required HLA-matched ESCs custom-tailored for this individual and thus circumvents immunogenicity issues. However, SCNT remains technically demanding, cost-inefficient, and ethically disputable. Manipulations of human embryos as well as ethical and legal concerns related to potential human cloning are restricted. Therefore, there is a need for patient-derived PSCs and differentiated cell types applicable for cell-replacement therapies, which offer the advantage to circumvent immunological problems without being considered as ethically and legally concerning [15].

In 2006, Takahashi and Yamanaka discovered that mouse embryonic and adult fibroblasts could be reprogrammed to cells with the characteristics of ESCs by overexpression of a defined set of ESC-enriched transcription factors (Oct4, Sox2, Klf4, and c-Myc). The resulting cells, termed iPSCs, display infinite self-renewal ability (stemness) and can differentiate into all three embryonic germ layers (pluripotency) [2]. Shortly after this seminal discovery, several laboratories reported the successful generation of iPSCs from diverse organisms and tissue types, including humans [1, 2, 19, 20].

iPSCs, sharing many of the regenerative properties of ESCs, are an invaluable source for regenerative medicine and hold great promise as a therapeutic product to help treat many overwhelming and life-threatening diseases that are currently incurable. Indeed, several iPSC-based clinical trials have recently been underway to treat macular degeneration, Parkinson’s and heart disease, highlighting the rapid progress that continues to be made in this area [6, 21]. Thus, ethical, legal, and safety considerations for the use of these cells are of crucial importance and should be counted in line with research and development. The present review explores the ethical, legal, and social aspects of iPSC generation and application from various points of view, discusses accepted regulations, poses key questions, and provides suggestions for researchers, legal authorities, and social activists in the field of iPSCs.

## Human iPSCs: methods of generation and biomedical applications

**Fig. 1:**
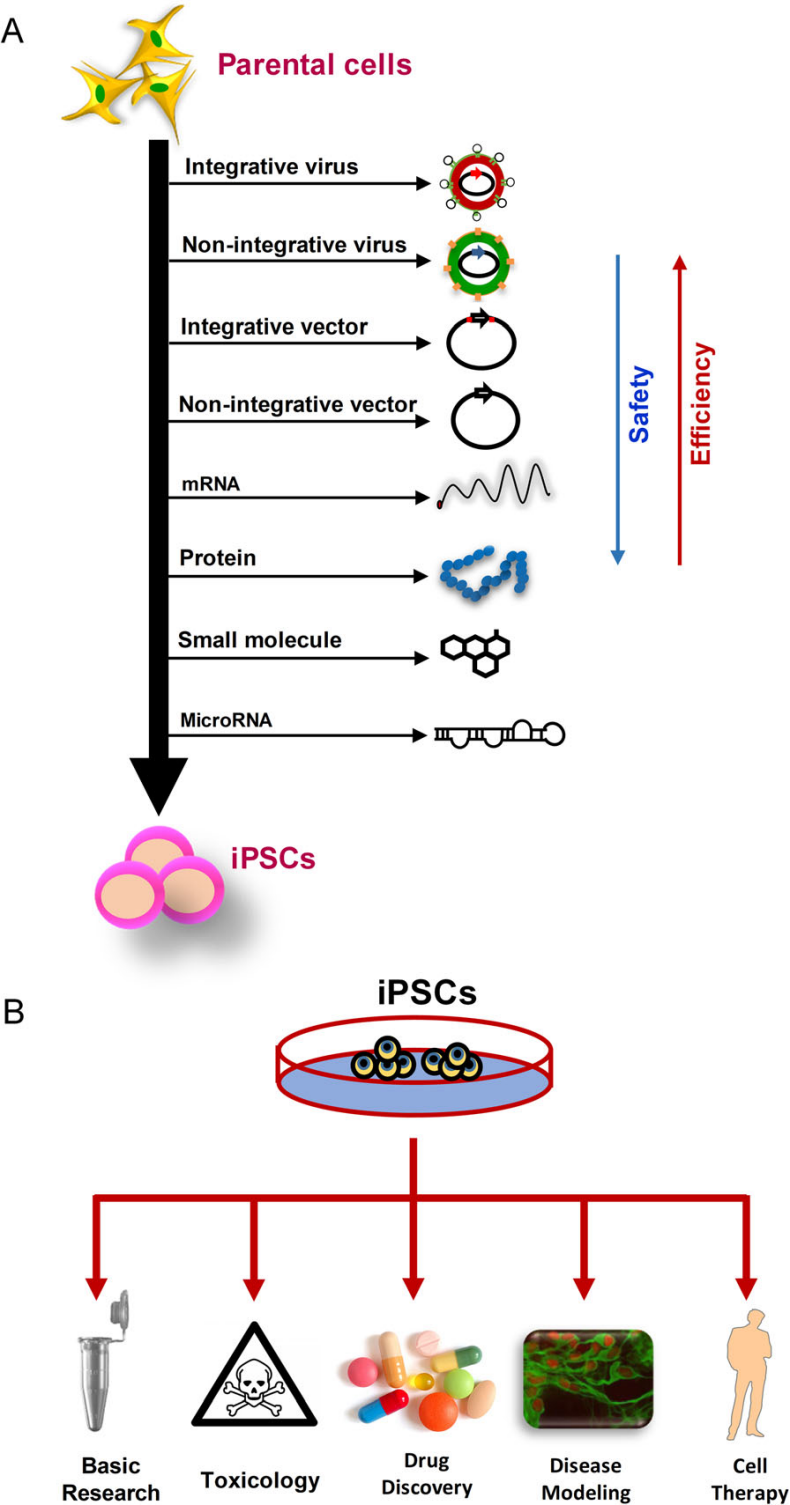
Generation and applications of iPSCs. **a** Various methods and approaches are used to convert somatic cells into iPSCs. Integrative methods such as integrative viruses and vectors provide the highest reprogramming efficiency but the lowest safety. In contrast, non-integrative approaches such as the use of small molecules and microRNAs tend to have a less reprogramming efficiency. Notably, episomal vectors, which do not integrate with the host cell’s genome, appear to provide both a high efficiency of iPSC generation and sufficient degree of safety. Although all the illustrated approaches could potentially be used to produce iPSCs for applications such as basic research, drug screening, and disease modeling, genomic integration should be avoided for generation of clinical-grade iPSCs. **b** Because of immortality and immense differentiation potential, iPSCs have all the potential biomedical applications of ESCs. They can be used to model pluripotency and multi-lineage differentiation in vitro, screen and discover new drugs, and establish disease models in a dish. iPSCs also hold a great potential to be used for replacing diseased or lost tissues, which needs specific considerations to provide safe, clinical-grade cells for transplantation into patients

Human iPSCs are a promising prospect for cell therapy in a wide range of diseases for which there are currently no cures or effective therapies, such as neurodegenerative diseases of the central nervous system, heart infarction, diabetes mellitus, and diseases of the liver, lung, and kidney. Given that iPSCs can be produced in a patient-specific manner, they may be used in autologous transplantation—avoiding complications of rejection by the host immune system. Various methods have been adopted to induce pluripotency in somatic cells which can be categorized into integrative approaches, in which foreign DNA sequences encoding reprogramming factors are inserted into the genome of the starting cells, and non-integrative methods, which do not require permanent genetic modification [6, 22] (Fig. 1a). Although iPSCs generated using integrative methods can be effectively used to conduct basic studies, discover new drugs, screen toxins, and model diseases in vitro, non-integrative methods offer the significant advantage to potentially produce “safe” iPSCs in which the potential of acquiring secondary disease-causing mutations is minimized and are thus considered to be more suitable for cell-based therapies.

Generation of safe, clinical-grade iPSCs through episomal vectors is such a non-integrative approach and is routinely employed by the Center for iPS Cell Research and Application (CiRA) at the Kyoto University in Japan, the first center dedicated to develop iPSCs for scientific and therapeutic applications [22]. Although the integrative approaches have nowadays become almost obsolete as a method for generation of new iPSC lines for therapeutic use, stable genetic modification of iPSCs and their derivatives may still play a significant role in the therapeutic context in order to fix a genetic, disease-causing problem and/or enhance the functionality, viability or other properties of cells used for transplantation [23–25]. Therefore, some of the considerations concerning the genetic manipulation of cells are also discussed in this article.

Because of immortality and multi-lineage differentiation potential, iPSCs are equally suitable for all the potential biomedical applications of ESCs (Fig. 1b). Viruses and/or integrative methods usually have the highest reprogramming efficiency but the lowest degree of safety (e.g., insertional mutagenesis and presence of viral components). In contrast, safer approaches such as the use of small molecules (e.g., RepSox, valproic acid, and tranylcypromine [26–29]), microRNAs (e.g., miR-302~367 cluster, miR-371~373 cluster, miR-17 family [30–34]), or metabolites (e.g., sodium butyrate, ascorbic acid, and forskolin [29, 35, 36]) either have less reprogramming efficiency or usually cannot induce pluripotency alone and are therefore frequently used in combination(s) with classical reprogramming factors [29, 37, 38]. Transfection of mRNAs that code for the classical reprogramming factors have also been used to induce pluripotency in somatic cells [39, 40], but due to their inherent instability, the mRNAs need to be transfected repeatedly. This is not only labor intensive but it also brings the risk that the mRNAs might be reverse-transcribed into DNA and integrated into the genome of the transfected cells. Genomic integration may lead to disruption of tumor suppressor genes and/or aberrant permanent activation of proto-oncogenes, thereby potentially giving rise to the malignant transformation of the genetically modified cells. This is particularly true when retroviruses are used, since they tend to randomly integrate into the host cell’s genome. Alternatively, the pluripotency genes used to generate iPSCs (in particular, the proto-oncogenes *c-MYC* and *KLF4*) may later become re-activated in the transplanted cells differentiated from iPSCs [41]. The safety of the reprogramming strategy used for iPSC establishment is not a significant concern when iPSCs are used for applications other than regenerative medicine. Therefore, integrative methods, which offer the advantage of high reprogramming efficiency, could be employed in disease modeling, drug screening, and basic research. Of note, non-integrative approaches have nowadays become much more popular and widespread than before in most iPSC laboratories. Even for non-regenerative uses, it seems much better to use iPSCs generated with non-integrative approaches because integration of the transgene might affect the behavior of iPSCs or their derivatives and therefore render them rather unsuitable even for in vitro applications. The in vitro generation, expansion, and differentiation of iPSCs can lead to detrimental epigenetic aberrations and/or genetic mutations which might occur as an artifactual result of culture adaptation [42–44]. Such abnormal epigenomic or genomic changes can affect growth and/or differentiation propensities as well as the functionality of iPSC lines, and hence their utility for downstream purposes [44–46]. Therefore, significant preference lies in using reprogramming (and differentiation) strategies that exhibit faster kinetics in order to minimize culture-induced epigenetic and genetic changes. For regenerative purposes, the low reprogramming efficiency could be alleviated by combining methods such as using microRNAs and small molecules together or applying alternative non-integrative vectors such as episomal vectors which can potentially provide both high safety and significant efficacy [25]. Due to their self-renewal ability, PSCs can be propagated indefinitely; hence, even if the method used to generate iPSCs has low efficiency, it is possible to select and expand a small number of the high-quality iPSC colonies, thereby providing high-quality lines of iPSCs for potential use in cell-replacement therapy. In cases where iPSCs are derived from a patient with a certain genetic defect, the genetic problem could be corrected prior to transplantation using special genome-editing tools such as a clustered regularly interspaced short palindromic repeats/CRISPR-associated 9 (CRISPR/Cas9) system [47, 48]. CRISPR/Cas technology is a newly developed, powerful tool for precisely altering DNA sequences and modulating protein (and therefore cellular) function. Due to its simplicity, efficiency, and affordability, the CRISPR technology is rapidly becoming more popular than other more technically demanding and time-consuming genome-editing approaches, such as the previously used zinc finger nuclease (ZFN) and transcription activator-like effector nuclease (TALEN) technologies, although its unwanted effects on the genome should be carefully taken into consideration [49]. With the advent of CRISPR, one can even revert disease-causing mutations in disease-specific iPSCs and differentiate and then transplant the genetically modified cells to replace the damaged or diseased tissues. Therefore, in combination with CRISPR technology, iPSC technology has even more potential in regenerative medicine. Overall, iPSC technology has offered an unprecedented opportunity to tackle devastating diseases which cannot be cured using available medical interventions, highlighting the need to understand how they can be effectively applied to treat patients.

## Ethical, legal, and social issues in using iPSCs for therapy

Many guidelines for human experimentations such as the Nuremberg Code (1947) and the related Declaration of Helsinki as well as the Belmont Report (1978) have been put forward years ago to restrict unethical research and therapy with human subjects. Notably, the latter two guidelines are widely regarded as the cornerstone documents on human research ethics today. Although these guidelines still apply today, things have changed and new possibilities have emerged. Thus, they need to be revisited and changed according to the advancement of technologies that impinge on the current ethics of today’s rapidly changing society. A major challenge is/will be, however, that such ethical rules must be acknowledged and abided by on global terms and not only in individual countries that all make up their own rules and ethics. Therefore, with the advancement of technology in the field of biomedicine and the emergence of new fields such as stem cell research and genome editing, these new technologies require a set of new specific rules to be included in the regulations to enable their application in these broad fields, and specifically in the area of regenerative medicine.

Given that in cell therapy, the cells are injected into patients as a live component with complex features and functions, applying the same regulations of drug therapy to this topic is not feasible. Therefore, a separate set of rules and conditions is required for using cells, particularly stem cells, in cell-replacement therapies. There are currently several guidelines for the use of cell, tissue, and stem-cell products in treating patients, most notably the US Food and Drug Administration (FDA) [50] and the European Medicines Agency (EMA) guidelines (https://www.ema.europa.eu/documents/scientific-guideline/guideline-human-cell-based-medicinal-products_en.pdf). Specialist associations such as the International Society for Stem Cell Research (ISSCR) have also separately developed or updated specific guidelines for the use of stem cells in cell therapy by the help of experts from all around the world [51]. These guidelines share many important outlines and differ from each other mostly in minor issues. According to them, the most important topics related to ethical, legal, and social considerations of cell therapy include (i) manufacturing conditions and characterization of clinical-grade cells, (ii) genetic material and confidential personal information, (iii) informed consent, (iv) genetic manipulation of the cells, and (v) intellectual property and patents, along with some other important issues.

### Manufacturing and characterization of clinical-grade cells

Legislative authorities have set the terms and conditions for producing any chemical and biological products or devices that are used to treat human diseases, the best and most applicable of which being Good Manufacturing Practices (GMP). GMP is a set of conditions that define the principles and details of the manufacturing process, quality control, evaluations, and documentation for a certain product [52]. At present, several organizations and authorities have issued GMP principles for pharmaceutical products to be used as guidelines, most notably the FDA, EMA, World Health Organization (WHO), International Conference of Harmonization (ICH), and Pharmaceutical Inspection Co-operation Scheme (PIC/S). The overall principles of GMP guidelines published by these institutions are compatible with each other. The most important issues addressed in the GMP principles are (i) the facility and equipment design in a way that enables the control of the procedures, (ii) adequate and precise documentation, (iii) control of production and processes, (iv) quality control and assurance, (v) validation, (vi) equipment calibration, (vii) personnel training and certification, and (ix) environmental monitoring (in terms of environmental and microbial contamination) [53]. In GMP, controls are risk-based, and since cell therapy is more complex than other therapeutic approaches such as drug therapy, there will be more risks in its production. Therefore, more control is needed for cell-based therapies. Similarly, cellular products in the category of Advanced Therapy Medicinal Products (ATMPs) require even more control (than non-ATMP therapies). ATMPs refer to biological products that have undergone specific manipulations during their manufacturing process, or, if left unmanipulated, can be of non-homologous use [54]. Since iPSC generation requires specific cellular (and genetic) manipulations, therapeutically relevant cells differentiated from iPSCs should undergo rigorous production process controls and documentation according to the GMP principles for ATMPs. Important aspects in GMP production of ATMPs include the clarification of clean room conditions, monitoring and safety, workflow, storage and biobank establishment, and the management of equipment, water, and waste materials [55]. The ATMPs’ quality control tests aim to monitor and ensure that there are no hazards in the four main features of an ATMP including safety, identity, purity, and potency [56]. In this way, the properly defined use of iPSC derivatives for therapeutic purposes requires the implementation of a series of risk-based controls and compliance with the GMP principles for ATMPs, so that the resulting cellular product derived from iPSC reprogramming can be properly applied for disease treatment. Importantly, ATMPs are needed to be validated for quality consistency and successful demonstration of manufacturing. According to recent GMP-ATMP guidelines, for investigational ATMPs, analytical experimental procedures do not need full verification, whereas clinical (also called authorized) ATMPs (clinical products that have reached the marketing authorization) require full validation in advanced experimental phases (https://ec.europa.eu/health/sites/health/files/files/eudralex/vol-4/2017_11_22_guidelines_gmp_for_atmps.pdf).

### Genetic material and confidential personal information

Like any cell, iPSCs derived from any individual will inherently contain a vast amount of private information (DNA) which, if used carelessly, may violate law, morality, and privacy of individuals. Even if the starting cell donor is not alive, the iPSCs contain his/her close relatives’ information, hence potentially bringing about ethical and legal challenges related to individual privacy [57]. This problem cannot be resolved by clearing the donor’s identity information at the time of donating the cells, because the genetic information might be sufficient to identify the donor or the donor’s relatives due to increasing availability of human genome sequencing data through public and private platforms [58, 59]. In addition, complete abolition of the donor’s information is often not desirable, because subsequent research on iPSCs may necessitate ongoing access to the information about the donor’s health status, requiring the knowledge of the donor’s name and address. In the course of data analysis within a specific project, researchers may also coincidentally discover that the donor might unknowingly suffer from a certain type of genetic disease. Rules of ethical conduct in many countries prohibit researchers to reveal such information to employment agencies, employers, third-parties, and even patients themselves without their consent. Therefore, it is imperative that appropriate actions are taken to not only prevent public disclosure of patients’ private information but also its disclosure to patients themselves if they choose the right not to know. Notably, these issues are similarly valid for any other cell type isolated from patients or healthy individuals, highlighting the importance of ethical considerations in this regard. In recent years, the use of anonymous social media such as Whisper and Secret along with less-anonymous social media sites such as Instagram, Facebook, and Twitter (provided that the users properly manage their privacy settings) has greatly facilitated the networking capabilities which has aided in recruiting patients but also permit to maintain a degree of anonymity to protect their privacy and avoid or reduce physical presence [60–63]. Despite the growing popularity and benefits of social media for recruiting participants as well as for making informed decisions (for example, about participating in a clinical trial or not), such media may endanger the normal flow of clinical studies when, for example, patients receiving iPSC-based cell therapy (or their parents) publicly disclose the results of clinical trials or discuss potential adverse or beneficial effects of the therapy without professional guidance, thereby potentially influencing the results of the trial and impairing its integrity [61, 64]. Overall, the growing prevalence of social media for such activities has both advantages and disadvantages which should be taken into consideration when recruiting patients for clinical studies.

### Informed consent in research and therapy with iPSCs

Whenever it is planned that humans or their cells and tissues are to be used in research projects, it is mandatory to take informed and voluntary consent from the participants [65]. The kind of informed consent form and the details within it are important. For instance, if the patient-derived iPSCs are supposed to be used only for basic laboratory research, this should be mentioned in the consent form and disclosed to the cell donor [66]. The consent form and the content of informed consent are proposed by the principal investigator, and after careful review, it is approved by the institutions’ ethics committees and/or regulatory authorities. Generally, one of the involved researchers who is aware of all the content of the research should explain the essence and purpose of the research to each participant (or, in cases where the patient is not able to make a decision, to the patient’s custodian) and then discuss with the recipients the possible side effects of the treatment, if the cells are intended to be used for therapeutic purposes. In fact, the participants’ role in the course of treatment should be defined in a plain language with complete details to the patient. This is usually prepared as a separate written document which is handed over to the study participant prior to his/her actual participation so that the participant has sufficient time to review all the information and to ask questions if needed. Patients should be aware of their rights, duration of the study, circumstances for their withdrawal from the study, risks, and number of participants. It is also required to describe all the therapeutic options to the participant and to answer all the patient’s questions. Therefore, researchers are not allowed to carry out other types of research or actions with the donor’s iPSCs in cases that informed consent was obtained only to perform basic research with their cells in only a specific project. However, if researchers want to contact the cell donor if they need to conduct additional studies with their iPSC lines in the future, the consent should contain the statement in which the participant can decide whether he/she agrees to be contacted or not as well as the declaration stating that the iPSC lines derived from the donated cells can be used in future research projects that still cannot be specified at the time of tissue procurement. If the study participant consents to this clause, the researchers have greater flexibility to use the cells in different projects. The donor may also oppose the production of germ cells from his/her iPSCs, and ignoring this right by researchers would inevitably entail ethical and legal challenges [57, 67, 68].

According to the conventional standards of research ethics, individuals can refrain from participating in the research project at any time, and even if embryos, for example, have already been derived from their cells, they could request to destroy them. However, since the derivation of iPSCs from donated cells is a very costly and time-consuming process, it would be detrimental if the withdrawal at a particular stage of the research project would be possible [69], because it would lead to the waste of time and resources. In addition, it would conceivably decrease the meaningfulness of conclusions drawn from a clinical study. Thus, a rigorous discussion of such situations is clearly warranted. Since in many cases the treatments may lack sufficient efficacy and/or elicit serious side effects in patients, it would be inevitable for patients to withdraw their consent during clinical studies. Therefore, the patients’ right for withdrawal at some time during the clinical study should be recognized. In other situations where patients do not explain the reason for discontinuing their participation in the clinical study but their withdrawal negatively influences the validity of the study, the researchers should explain to the patients the critical importance of their participation in the study for the completion of the clinical trial. It is important that such issues are clearly stated in the consent form and that the time period in which the donor still can withdraw from the study and request for destruction of her/his cells is clearly specified without major consequences [70]. However, it might not be always possible to change consent if, for example, the cells have been used, or are needed, as quality controls for existing treatments and/or ongoing drug discovery purposes. Therefore, clear policies must be in effect to remove the unnecessary barriers in doing research and respect the rights and privacy of individuals.

An individual, from whom iPSCs have been derived, may want to know about the fate of his/her iPSCs (in terms of research and commercial aspects). Do donors have the right to expect financial gain from the commercial benefits of their cells? It should be taken into consideration that if donors are offered financial award for the participation, the participation can no longer be called voluntary. Another question is whether the donors have the right to control and direct the derived iPSCs or their products in future. In fact, any reasonable use of iPSCs would be very difficult if the donor of the original somatic cells would have control over the fate of iPSCs, after a specified period of time; otherwise, it would greatly limit the researchers’ freedom of operation. The existence of international differences in legislation, jurisprudence, law, and philosophical approaches greatly intensifies the complexity of such legal cases [57].

It should be borne in mind that although the derived iPSCs are genetically identical to the somatic cell donor, the cells have been modified such that they have little structural, functional, and epigenetic similarity to the donor’s primary cells [25, 71]. The question now arises whether this technical fact (changing the identity of the cell) can give donors the right to have control on the cells’ usage [57]. Overall, all these facts and challenges need to be put together to make the right decision about the legal scope of individuals to control their products, as well as to carry out fundamental and applied research on humans.

It is essential to obtain informed consent from patients who are scheduled to undergo iPSC-based clinical trials (i.e., patients in both treatment and control groups) [72]. In the case of randomized, controlled clinical trials, patients should be told that their chance of being in the control group is randomized. In addition to the oral explanation, this information must be presented in written form to each patient, with a signed form serving as a document. The most important difference between stem-cell-based clinical trials and drug-based clinical trials is that in cell therapy, patients should be provided with sufficient evidence and information about the identity and cellular potency of the administered cells, as well as ensured that the same cells which have been preliminarily approved in a pre-clinical study will be used in the clinical trial [73].

### Considerations for the use of genetically modified cells

In cases where the purpose of iPSC generation is using the cellular derivatives for the treatment of diseases, the reprogramming approach should be as safe as possible. Therefore, non-integrative methods can be used to simultaneously provide an appropriate level of safety and a sufficient reprogramming efficiency. The resulting clinical-grade iPSC line should be carefully evaluated for genomic and karyotype integrity. It may also be needed to evaluate iPSCs and their cellular derivatives through genome-wide DNA sequencing to determine if there are any disease-causing mutations.

Although the non-integrative methods are the preferred strategy for safe iPSC generation, it is possible that in some specific cases (e.g., to genetically correct a genetic disease), integrative vectors or viruses will be used. Notably, due to safety concerns of applying genetically modified iPSCs in humans, regulatory agencies have always had a strict inspection policy for using transgenic cells for patient treatment [74]. The recent approval of several transgenic cell products has opened up new horizons for gene therapy and led to conducting numerous clinical trials around the world for the investigation of various gene therapies with different gene-editing tools such as TALEN and CRISPR/Cas9 systems [75–78]. The most important difference, in terms of GMP guidelines, between transgenic cell therapy and cell therapy with genetically unmodified cells is to ensure the safety of the transgenic cells. Genetic manipulation of the therapeutically relevant cells using tools (e.g., retroviral or lentiviral vectors) randomly inserting a gene of interest into the genome, will have the highest safety risks, thereby requiring even more quality controls to avoid undesired effects on the therapeutic potency and safety profile of a cell-based product. Non-random integrative methods such as transposon-based systems and targeted gene-editing approaches based on ZFN, TALEN, or CRISPR/Cas9 technologies also require appropriate quality control tests, due to the possibility of off-target mutagenesis [79]. The genetic modification of iPSCs might be needed, for example, to repair disease-causing mutations or replace an entire gene/exon in genetic-disease-specific iPSCs when doing autologous iPSC-based cell therapy. iPSCs or their derivatives could also be genetically modified in order to enhance the properties of native cells to, for example, improve their therapeutic potency by increasing their survival upon transplantation or by enabling the cells to secrete additional factors that could induce endogenous regenerative processes in addition to effects exerted by transplanted exogenous cells.

It is worth mentioning that one of the key challenges in producing genetically modified iPSCs for cell therapy is the production of clinical-grade viral vectors. The production of such vectors under GMP conditions requires special equipment and facilities as well as highly experienced and skilled operators [80–82]. Therefore, the safety of clinical-grade vectors must be confirmed to provide the highest level of reliability and patient safety.

### Patent and intellectual property

Intellectual property defines non-financial rights and assets which originates from mental activities and creativity and includes, among others, copyright, patents, trademarks, and artistic and literary works. Since the intellectual property objective is to provide intellectual goods, laws have been set up to protect inventions and grant the rights for products to individuals, creators, and inventors for a limited period of time. Although initial attempts to produce ESCs were patented, it has been debated that the patents which require the destruction of human embryos for ESC generation should not be accepted anymore. Potentially, iPSC technology may also be subjected to patent barriers. Some patent organizations oppose the idea that ESCs and iPSCs are considered the same in terms of entity and, therefore, their patent process. Although the technology for generation of iPSC lines was rightfully patented by Shinya Yamanaka who first generated these invaluable cells, it has not yet been clarified whether the various (slightly) modified methods for generation of iPSCs can be similarly patented or if they lack sufficient novelty. If a previously patented technique for induction of pluripotency in one somatic cell type is applied to another cell type, can the resulting iPSCs be patented? Although it has been reported that different lines of iPSCs (and ESCs) have different expression patterns as well as different characteristics, these differences appear to be negligible when it comes to comparing a larger number of iPSC (and ESC) lines [22, 83]. As a result, it would be important to determine the extent to which features and differences in iPSC lines or reprogramming strategies can be considered patentable.

Patents are valuable not only for the progress and maturation of science but also for the commercialization and clinical application of the reprogramming technology. However, if several patents held by different inventors are essential to advance science and technology and translate basic research into commercialized or clinical products, this might potentially frustrate the rapid clinical translation of basic research and would probably limit the researchers’ creativity. Therefore, further debate and research is needed to adjust the gap between patent and innovations.

## iPSCs: potential for human cloning, germ cell production, and beyond

Despite the ethical advantage of iPSCs over ESCs, there are still significant concerns regarding the ability of iPSCs to be used for production of interspecies chimeric animals, human reproductive cloning, or generation of human gametes [84–86]. Although many of these ethical concerns have already been raised about ESCs, the ease and simplicity of obtaining starting cell sources for iPSC generation together with the fact that these cells might be obtained even without donor consent, highlight the need to apply specific rules in this regard.

### Production of animal models

iPSCs can be generated from various domesticated and farm animals [87–90]. Production of iPSCs from domesticated animal species and companion animals such as dogs, cattle, chickens, and pigs is economically valuable and critically important for the establishment of disease models as well as the production of medically useful substances, e.g., enzymes and growth hormones, which are absent or inadequate in patients suffering from specific genetic diseases. Importantly, iPSCs themselves or cells differentiated from iPSCs (particularly cardiomyocytes and hepatocytes) can also be directly used for disease modeling and drug screening [91–94], thereby significantly decreasing the extent to which animals are used for research purposes. More recently, iPSCs have been harnessed as a potential means to reduce animal slaughtering by serving as an immense cell source for large-scale production of cultured meat. Using iPSC technology, umbilical cord blood cells obtained from cattle after delivery can be reprogrammed to iPSCs, which can subsequently be differentiated into lab-grown muscle and fat cells [95, 96], reducing the need to sacrifice animals. The main challenge about this uprising technology is whether meat derived from iPSCs tastes like meat derived from animals and whether iPSC technology to generate meat can be developed sufficiently to be economically cost-effective. Notwithstanding, if proven safe and ethical, generating meat from iPSCs might prove to be highly advantageous, since they can self-renew for long term in culture and efficiently differentiate into both muscle and fat cells. In addition, the iPSC technology has the potential to be applied for rescuing endangered animal species in the future [97]. iPSCs could also be used for the generation of humanized organs inside large animals through interspecies blastocyst complementation. In this technique, animals consisted of cells from animal and human are generated by microinjecting human iPSCs into animal blastocysts. Genome editing tools are used to ablate essential genes for the development of a specific organ of interest, thereby permitting the donor iPSCs to colonize the vacated niche with negligible competition from the host [85, 98, 99]. In this way, a desired organ of human origin will be generated inside a host animal, thereby expanding human organ supply to address the dire shortage of organs for transplantation. The best animal hosts for growing human organs are considered to be large animals with high physiological similarity to humans such as cattle and pigs. Since pigs have a 16-week gestation, they might not be the best option for human organ regeneration, as human embryogenesis is 40 weeks. Cattle embryos, in contrast, normally develop for 40 weeks, potentially providing a better match for generating human organs. Of note, host animals chimerized with human iPSCs provide a reliable functional assay for confirming the full pluripotency of human iPSCs. Due to ethical concerns of human-animal chimerism, it would be important to develop in vitro functional assays (that are more informative than teratoma formation) in order to exclude or reduce the use of animals as hosts for testing the pluripotency of human PSCs (including iPSCs). For regenerating human organs, however, animal hosts appear to be the best available option, although it is ethically challenging. In fact, blastocyst complementation might potentially lead to the generation of acute human/non-human chimeras with an ambiguous moral status, because human iPSCs might differentiate into brain cells in the chimeric animals. In other words, acute human/animal chimerism might create animals that not only have humanized organs but are also morally humanized. Acquiring a human-like consciousness by chimeric animals is ethically unacceptable, since consciousness represents a major distinction between animals and humans. Moreover, the human iPSCs injected into the host animals might give rise to human gametes in the chimeric animals, posing a serious ethical conundrum. Another issue is that the humanized organs might be contaminated with cells from the host animal, bringing about immunologic and potential functionality issues upon transplantation into patients. A recent study, in which mouse kidney was grown in host rats through cross-species chimerism, observed that the chimeric rats with mouse kidney died shortly after birth, apparently because the genetic ablation of *Sall1* gene for generating a vacated niche in rat embryos had also removed their sense of smell, preventing them from detecting milk and subsequent suckling, leading to their death [100]. This finding highlights the notion that generation of a suitable host animal for growing a human organ might come at a price, compromising the survival of the genetically modified host animal and more importantly limiting the utility of the animal-hosted human organ. Another risk for patients needing animal-hosted human organs is that the endogenous viruses such as porcine endogenous retroviruses (PERV) and/or viral DNA in the animal host’s genome might then be re-activated in the patient body, causing potentially life-threatening infections or altering the behavior of cells within the humanized organ to be used for transplantation, disease modeling, or drug discovery. It is also possible that similar viruses/viral sequences originated from human iPSCs are transmitted to the host animal. Notably, if the ethical, safety, and functionality issues surrounding humanized organs developed in animal hosts are finally resolved, such organs would have a high cost at least at the beginning, which would restrict their availability to all patients, particularly if such products are not sufficiently supported by public health providers or insurances. Therefore, further research and debate are needed to properly address major ethical concerns associated with human/animal chimerism.

### Human reproductive cloning

The use of PSCs (including iPSCs), SCNT technology, or any other method for human reproductive cloning is prohibited, illegal, and punishable worldwide. Some technologies, particularly tetraploid complementation, could theoretically enable human reproductive cloning with iPSCs. Tetraploid complementation is a technique in biology used to produce fetuses entirely derived from PSCs, thereby serving as the most stringent method to confirm the pluripotency of different types of PSCs. In this assay, diploid PSCs are injected into tetraploid blastocysts (which are unable to give rise to an entire organism per se) to generate embryos fully derived from the diploid PSCs. Although this technique is most commonly used to obtain the so-called all-PSC mice as a strategy to rigorously characterize mouse PSCs or to establish all-PSC mouse models of a certain human disease, it might have the potential to be used for illegal human cloning. Human iPSCs (as well as human ESCs) are developmentally similar to murine epiblast stem cells which have a low ability to produce chimeric animals (and thus a poor ability to produce iPSC-only organisms via tetraploid complementation). Traditional (primed) human PSCs can be converted in vitro into the so-called naïve PSCs that are similar to mouse ESCs and thus might have enhanced ability for cloning (reviewed in [34, 101]). However, it is still not clear how efficiently naïve human PSCs might potentially contribute to the formation of human fetuses, as naïve human PSCs generated under different conditions do not display the full spectrum of naivety features [102]. Of note, recent achievement of the Chinese researchers who cloned the ape via SCNT [103] suggests that human cloning is not scientifically impossible nor far from reachable, as there are numerous similarities between humans and primates in terms of embryogenesis and physiology. This highlights the need to strengthen surveillance by regulatory agencies and maximize the ethical integrity of research projects dealing with SCNT and tetraploid complementation.

### Generation of human gametes from iPSCs

There are several important reports indicating that gametes could be generated from PSCs (including iPSCs). Hayashi et al reported that mouse ESCs/iPSCs could be differentiated into primordial germ cell (PGC)-like cells which could contribute to oogenesis and spermatogenesis and finally to healthy offspring when transplanted in vivo [104, 105]. Researchers have also reported the successful differentiation of human PSCs into PGC-like cells [106, 107] and human immature oocytes from iPSCs [108], suggesting that human PSCs could eventually be differentiated into fully functional human sperm and oocyte in vitro. Human iPSCs might be advantageous over human ESCs for reproductive biomedicine applications, since there are fewer ethical concerns associated with iPSCs and the sources for iPSC generation are abundant and more accessible. Moreover, iPSCs, as opposed to ESCs, could be easily produced from patient’s own cells and would not face immune rejection upon autologous transplantation of its germ cell derivatives. Therefore, the derivation of patient-specific gametes (in particular, sperm) from iPSCs would lay the foundation for the successful treatment of male infertility in the future.

The ability to produce human germ cells from iPSCs, despite all its hopes and benefits, is an ethical challenge, because the resulting germ cells may be illegally and immorally used for illicit reproductive practices. In this case, issues such as (i) informed consent, (ii) safety of the approaches used to generate and differentiate iPSCs, (iii) likelihood of human cloning, and (iv) the possibility of conceiving a child from an illegitimate donor would need to be realized and resolved. In addition, mature gametes potentially derived from human iPSCs (and ESCs) should be functionally evaluated before adoption, and this requires the production of early embryos, which is itself a controversial discussion. In countries such as the UK, Iran, and Singapore, production of human pre-implantation embryos for conducting basic studies is not ethically banned, but this is a barrier for most other countries. The ease with which iPSCs could be produced from individuals raises ethical concerns regarding the potential generation of human germ cells from PSCs in the future. Finally, the proper application of PSC-derived gametes and whether zygotes from hPSC-derived germ cells will be permitted to be used for research or therapeutic applications should be carefully taken into account from legal and ethical viewpoints.

## Conclusion

**Fig. 2:**
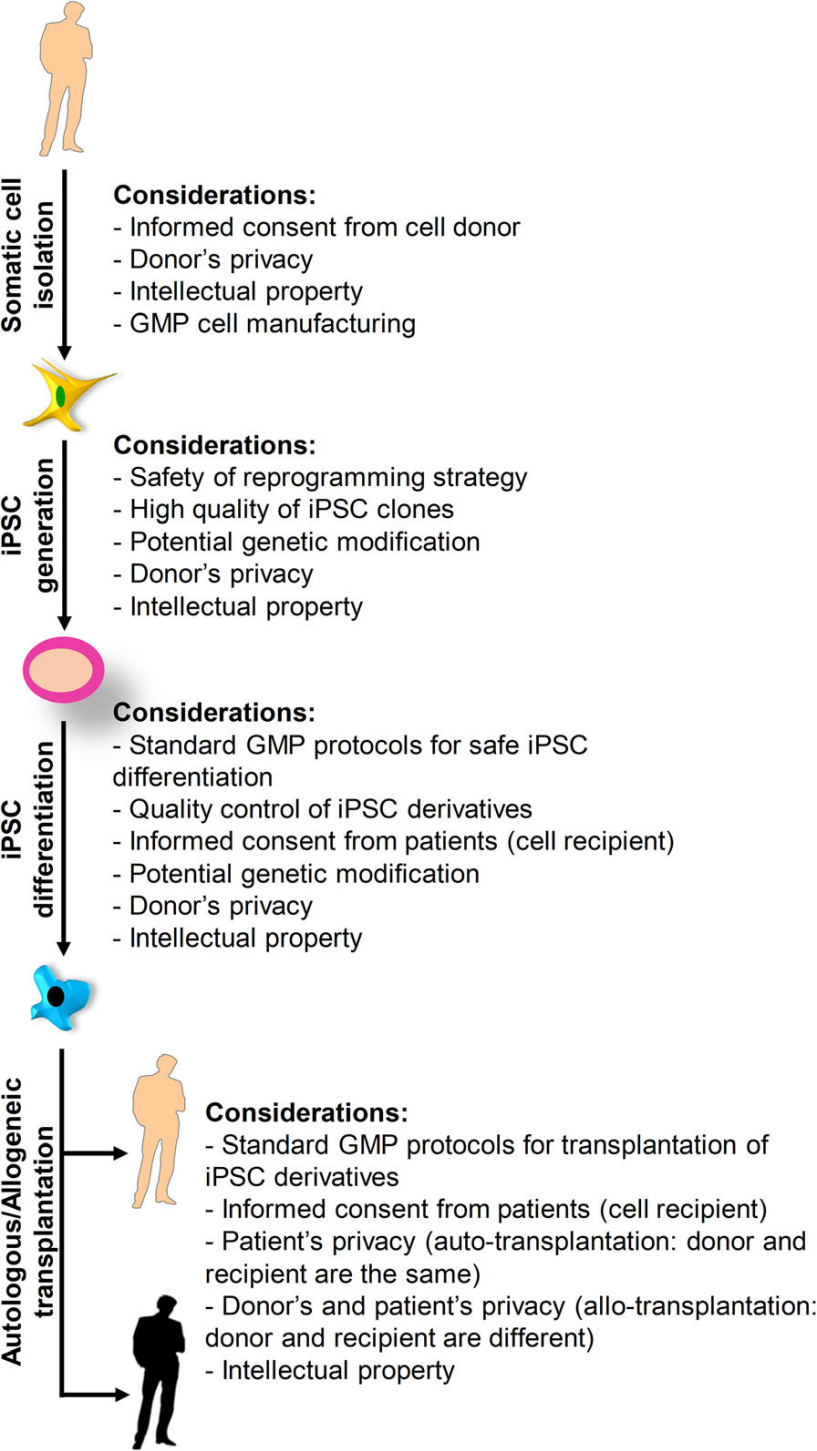
Ethical considerations of iPSC-based cell therapy. Ethical considerations apply to all the steps of iPSC-based cell therapy from somatic cell isolation to iPSC generation and differentiation to injection of iPSC derivatives into patients

iPSCs have opened up a new avenue for stem cell research and unique opportunities in the pharmaceutical industry and clinical practice. However, as with many other fields, reprogramming technology has its own ethical-social issues, all of which must be carefully considered. Laws and standards must be put in place to ensure the ethical integrity of iPSC production/application and to simultaneously remove unnecessary barriers in the way of research and therapy with iPSCs. In summary, informed consent must be obtained from both cell donor and recipient. Since the donated cells contain private information in the form of DNA, it must be ensured that the privacy of both donors and patients is protected. At the beginning of cell donation, donors should be made aware of the time period they may be able to have control on their cells. All the steps of iPSC-based cell therapy from somatic cell isolation from donor to iPSC generation and application to the transplantation of iPSC derivatives must be carried out under GMP conditions. The safety of the methods to derive or differentiate iPSCs is of significant importance. Specific quality control tests would be needed particularly if cells have undergone genetic manipulation (Fig. 2). Finally, the potential illegitimate usage of iPSCs for human cloning, generation of potentially acute human-animal chimeras, and illegal/unethical generation of human gametes from iPSCs must be borne in mind and further debated.

## Data Availability

Not applicable.

## Abbreviations

ATMP: Advanced Therapy Medicinal Product
CiRA: Center for iPS Cell Research and Application
CRISPR/Cas9: Clustered regularly interspaced short palindromic repeats/CRISPR-associated 9 system
ELSI: Ethical, legal, and social implications
EMA: European Medicines Agency
ESC: Embryonic stem cell
FDA: Food and Drug Administration
GMP: Good Manufacturing Practices
HLA: Human leukocyte antigen
ICH: International Conference of Harmonization
iPSC: Induced pluripotent stem cell
ISSCR: International Society for Stem Cell Research
PERV: Porcine endogenous retroviruses
PGC: Primordial germ cell
PIC/S: Pharmaceutical Inspection Co-operation Scheme
SCNT: Somatic cell nuclear transfer
TALEN: Transcription activator-like effector nuclease
WHO: World Health Organization
ZFN: Zinc finger nuclease

## Glossary

Tetraploid complementation: A procedure during which PSCs are injected into a tetraploid blastocyst-stage embryo. Only genuine PSCs are able to give rise to an entire organism through this technique, since the tetraploid blastocyst does not develop into the embryo proper but only to the extraembryonic fetal membranes.
Morula: A pre-implantation embryo consisting of 16 cells (also known as blastomeres) in a solid ball-shaped structure. It is developed from a zygote through rapid cell divisions and will give rise to the formation of blastocyst-stage embryo.
Blastocyst: An embryo with a thin-walled hollow structure at the pre-implantation stage containing a so-called inner cell mass which gives rise to the embryo and a single layer of trophectoderm cells contributing to the development of placenta.
Teratoma: A benign tumor containing various types of differentiated cells such as bone, tooth, hair, and many other cells. Direct injection of PSCs into immunocompromised mice will most commonly lead to the development of teratoma.
SCNT: An experimental procedure during which a nucleus of a somatic cell is injected into an enucleated egg cell, giving rise to generation of a viable embryo. The cytoplasm of the enucleated egg cell reprograms the transferred nucleus.
Cell fate reprogramming: Conversion of one cell type into another, which occurs most commonly in the lab.
Insertional mutagenesis: Insertion of foreign DNA into the cell’s genome to study gene function.
MicroRNAs: Short regulatory RNAs modulating gene expression at the post-transcriptional level, thereby contributing to the control of PSC behavior.
Tumor suppressor genes: Genes tending to restrict cell proliferation, thereby resisting cancer development.
Proto-oncogenes: Genes tending to stimulate normal cell proliferation, thereby promoting cancer cell growth when mutated. Mutated proto-oncogenes are called oncogenes.
Genome editing: Introduction of specific changes to the cells’ genetic material, i.e., DNA or genome, using specific molecular biology techniques.
Primed PSCs: PSCs exhibiting features of post-implantation embryos, e.g., higher differentiation leakage, high sensitivity to single cell dissociation, and lower proliferation rates.
Naïve PSCs: PSCs exhibiting characteristics of pre-implantation embryos, e.g., less differentiation leakage, insensitivity to single cell dissociation, and higher rate of proliferation.
